# Contribution of Serologic Assays in the Evaluation of Influenza Virus Infection Rates and Vaccine Efficacy in Pregnant Women: Report From Randomized Controlled Trials

**DOI:** 10.1093/cid/cix241

**Published:** 2017-03-21

**Authors:** Shabir A. Madhi, Marta C. Nunes, Adriana Weinberg, Locadiah Kuwanda, Andrea Hugo, Stephanie Jones, Nadia van Niekerk, Justin R. Ortiz, Kathleen M. Neuzil, Keith P. Klugman, Eric A. F. Simões, Clare L. Cutland, for the Maternal Flu Trial (Matflu) Team

**Affiliations:** 1 Department of Science and Technology/National Research Foundation, Vaccine Preventable Diseases and; 2 Medical Research Council, Respiratory and Meningeal Pathogens Research Unit, University of the Witwatersrand, and; 3 National Institute for Communicable Diseases, National Health Laboratory Service, Centre for Vaccines and Immunology, Johannesburg, South Africa;; 4 School of Medicine and Children’s Hospital, University of Colorado, Aurora;; 5 Department of Medicine and Department of Global Health, University of Washington, Seattle;; 6 Center for Vaccine Development, University of Maryland, Baltimore;; 7 School of Pathology, University of the Witwatersrand, Johannesburg, South Africa;; 8 Department of Pediatrics, Medicine and Pathology, University of Colorado School of Medicine, and; 9 Center for Global Health, Department of Epidemiology, Colorado School of Public Health, Aurora

**Keywords:** influenza vaccine, efficacy, phase III trial, immunogenicity, hemagglutination inhibition assay.

## Abstract

**Background.:**

The utility of serologic testing to evaluate vaccine efficacy of seasonal inactivated influenza vaccine (IIV) is controversial. We aimed to evaluate the efficacy of IIV against serologically diagnosed influenza infection (SDI) and reverse-transcription polymerase chain reaction–confirmed influenza illness (PCR-CI) in women vaccinated during pregnancy.

**Methods.:**

We undertook a post hoc analysis of 2 randomized clinical trials evaluating IIV efficacy among human immunodeficiency virus (HIV)–uninfected and HIV-infected pregnant women. SDI was defined as ≥4-fold increase in paired hemagglutinin antibody inhibition titers from 1 month postvaccination until end-of-study participation. PCR-CI was defined as molecular diagnostic evidence of influenza virus in pharyngeal specimens collected during clinical illness.

**Results.:**

Among placebo recipients, the respective incidence of PCR-CI and SDI was 5.6% and 35.0% in HIV-uninfected women and 20.5% and 43.6% among HIV-infected women. Vaccine efficacy in HIV-uninfected women was similar for PCR-CI (66.9%; 95% confidence interval [CI], –20.1% to 90.9%) and SDI (59.2%; 95% CI, 37.0%–73.5%); however, fewer women required vaccination to prevent 1 episode of SDI (5; 95% CI, 3–9) than PCR-CI (27; 95% CI, 12–∞). Also, vaccine efficacy was similar for PCR-CI (61.2%; 95% CI, 10.7%–83.2%) and SDI (60.9%; 95% CI, 33.9%–76.9%) in HIV-infected women, with 2-fold fewer women needing to be vaccinated to prevent SDI (4; 95% CI, 3–8) than PCR-CI (8; 95% CI, 4–52).

**Conclusions.:**

Although vaccine efficacy was similar when measured for PCR-CI or SDI, IIV vaccination prevented a greater number of SDI than PCR-CI; the clinical relevance of the former warrants interrogation.Clinical Trials Registration. NCT01306669 and NCT01306682

## Summary

Serology provided a more detailed evaluation of exposure to seasonal influenza virus. Vaccine efficacy was similar when measured for polymerase chain reaction–confirmed influenza illness (PCR-CI) or serologically diagnosed influenza infection (SDI) in pregnant women; however, vaccination prevented a greater number of SDIs than PCR-CIs.

Vaccination of pregnant women with seasonal trivalent inactivated influenza vaccine (IIV) is efficacious against influenza illness [1–3]. Vaccine efficacy against reverse-transcription polymerase chain reaction (RT-PCR)–confirmed influenza illness (PCR-CI) was 50% in human immunodeficiency virus (HIV)–uninfected women and 58% in HIV-infected women in a randomized controlled trial in South Africa, among whom the attack rate of PCR-CI was 4% and 17% in the respective placebo recipients [2]. Vaccination of pregnant women with IIV also protected their young infants (<6 months of age) against laboratory-confirmed influenza illness [1–6].

Although paired hemagglutination antibody inhibition (HAI) assays have been used in some studies to evaluate IIV effectiveness, the utility thereof in this context has been controversial since the 1960s [7–9]. Of concern is that serology as a measure of influenza virus infection could overestimate vaccine effectiveness, due to the high HAI titers induced by vaccination in IIV recipients, resulting in them being less likely to elicit a further 4-fold increase in titers following influenza virus infection (23%) compared with IIV-unvaccinated individuals (90%) [7]. Furthermore, serologically diagnosed influenza virus infection (SDI) may not necessarily manifest as a clinical illness and therefore might not represent an outcome of public health importance. Nevertheless, it could yield a better estimate of influenza virus exposure, which may be important to ascertain. For example, the magnitude of exposure of the fetus in utero to maternal influenza virus infection could affect influenza-associated adverse pregnancy outcomes such as prematurity or low birth weight [10, 11].

We report on a post hoc objective of a efficacy trial of IIV in separate cohorts of HIV-uninfected and HIV-infected pregnant women up to 24 weeks postpartum. Specifically, we evaluated the efficacy of IIV vaccination of pregnant women and the number needed to treat (NNT) against PCR-CI and/or SDI.

## METHODS

Details of the study cohorts in the 2 double-blind, randomized, placebo-controlled trials among HIV-infected and HIV-uninfected pregnant women, including the inclusion and exclusion criteria, have been published previously [2]. In brief, a cohort of 198 HIV-infected pregnant women was enrolled from 3 March to 2 June 2011, and 2 distinct cohorts of HIV-uninfected pregnant women were enrolled in 2011 (n = 1060) and in 2012 (n = 1056) at approximately 20–36 weeks of gestational age. While all HIV-infected women were included in an immunogenicity study on vaccine response and antibody kinetics, only a subset of HIV-uninfected women were included in the immunogenicity studies among the cohorts enrolled from 3 March to 24 June 2011 (n = 193) and 6 March to 11 June 2012 (n = 183). The women were randomized 1:1 to receive IIV or saline placebo. The influenza vaccine (Vaxigrip, lot number G05831 in 2011 and H7221-2 in 2012; Sanofi Pasteur, Lyon France) was commercially procured and was composed of the World Health Organization–recommended Southern Hemisphere vaccine strains (A/California/7/2009 [A/H1N1pdm09], A/Victoria/210/2009 [A/H3N2], and B/Brisbane/60/2008-like virus [B/Victoria]), which remained the same in 2011 and 2012.

Weekly surveillance of the participants was undertaken by home visit or telephone call to elicit the presence of respiratory and other symptoms through 24 weeks postpartum for both groups. Furthermore, weekly SMS (text) messages were sent to participants via mobile phone reminding them of the symptoms of influenza-like illness (ILI), as well as requesting them to attend the study clinic should they have ILI symptoms. Surveillance was also undertaken for all-cause and respiratory-associated hospitalizations, and participants were advised to attend the study clinic for any other intercurrent respiratory illness. All hospitalizations and unsolicited illness visits were similarly investigated by RT-PCR for influenza virus.

### Sample Collection and Investigation for Influenza Virus Infection

The methods used for identifying influenza virus among the women involved obtaining an oropharyngeal and nasopharyngeal swab using a flocked-tip plastic shaft swab (Tool and Carbide Plastics, South Africa) as described elsewhere [2]. Testing was undertaken by a qualitative 2-step RT-PCR assay. Primers and probe sets that target either the matrix gene or the hemagglutinin gene designed for the universal detection of type A and B influenza viruses, respectively, were used [12]. All influenza A viruses were further subtyped as either H1 or H3 and the B virus lineages determined as either B/Victoria (homotypic vaccine strain) or B/Yamagata [13].

### Blood Samples, Hemagglutination Inhibition Assays, and Serologic Endpoints

Blood samples from women in the immunogenicity study were obtained by venipuncture prior to study-allotted intervention administration and 1 month thereafter. Additional blood samples were taken within 7 days of delivery and 24 weeks postpartum. HAI assay was undertaken at the University of Colorado, as described [14]. SDI in women was defined as ≥4-fold increase in HAI titers to a specific vaccine strain, which was investigated on blood samples obtained at delivery and at 24 weeks postpartum compared with the previous specimen spaced at least 21 days apart, irrespective of presence of clinical illness. We also analyzed the rate of ≥4-fold increase in HAI titers between 1 month postvaccination and delivery visit, and between the latter time-point until 24 weeks postpartum for specimens spaced at least 21 days apart in the same individual.

### Statistical Analysis

Vaccine efficacy calculations were limited to participants who were included in the immunogenicity cohorts, which included the full HIV-infected cohort from 2011 and a total of 376 HIV-uninfected women from 2011 and 2012. All PCR-CI episodes in these women from enrollment to 24 weeks postpartum were included in the analyses. All women included in the current serologic analyses had at least 2 blood draws from 1 month following vaccination to 24 weeks postpartum.

For vaccine efficacy (VE) endpoints, participants were censored after the first episode for the specific outcome. The evaluated VE endpoints were PCR-CI, SDI based on ≥4-fold increase in HAI titer, and the composite of these endpoints. Because the exact timing of infection for SDI was not ascertainable, only a modified intent-to-treat analysis was undertaken, which included SDI cases from 1 month postvaccination (to exclude any immune response to vaccine as the cause of serologic conversion among the vaccinees) to 24 weeks postpartum.

Vaccine efficacy was calculated using the formula 1–I_V_/I_P_, (I_V_ = case incidence rate in the vaccinated group; I_P_ = case incidence rate in the placebo group); 95% confidence intervals (CIs) were constructed and differences between the intervention groups tested. We calculated the NNT for PCR-CI and SCI using the formula 1 / I_P_–I_V_. Agreement between serologic conversion (≥4-fold increase in HAI titer) and PCR detection was assessed by Cohen κ.

Study data were collected and managed using Research Electronic Data Capture [15]. All statistical analyses used Stata software version 13.1 (StataCorp, College Station, Texas). All *P* values were 2-sided and values <.05 were considered significant.

### Ethical Considerations

The studies (ClinicalTrial.gov numbers NCT01306669 and NCT01306682) were approved by the Human Research Ethics Committee (HREC) of the University of the Witwatersrand (HREC numbers 101106 and 101107) and conducted in accordance with Good Clinical Practice guidelines. Written informed consent was obtained from all participating women, including on behalf of their infants.

## RESULTS

Three hundred twenty-one of the 376 (85.4%) HIV-uninfected women enrolled in the immunogenicity cohort were included in this analysis. The disposition of the HIV-uninfected women and availability of samples for HAI serology testing is illustrated in Supplementary Figure 1*A*. Among the HIV-uninfected women included in this analysis, there were no differences between the IIV and placebo recipients at enrollment in mean age (26.4 years), mean body mass index (28.7 kg/m^2^), mean gestational age at enrollment (27.0 weeks), and percentage who had been previously pregnant (70.1%) (Table 1).

**Table 1.** Baseline Demographic Characteristics for the Human Immunodeficiency Virus–Uninfected Participants Included in the Immunogenicity Subset Cohort and Scheduled Visits Time-points

Characteristic	Overall	IIV	Placebo	*P* Value
Mean age, y (SD)	26.4 (5.4) [321]	26.4 (5.4) [161]	26.4 (5.4) [160]	.977
Mean body mass index, kg/m^2^ (SD)	28.7 (5.6) [259]	29.4 (5.8) [127]	28.1 (5.4) [132]	.056
Mean gestational age, wk (SD)	27.0 (4.4) [321]	26.9 (4.4) [161]	27.1 (4.4) [160]	.790
Nulliparous, No. (12)	126 (39.3) [321]	63 (39.1) [161]	63 (39.4) [160]	.964
Primigravida, No. (12)	96 (29.9) [321]	52 (32.3) [161]	44 (27.5) [160]	.348
Mean days after vaccination of first postvaccination immunogenicity visit (SD)	30.4 (5.4) [306]	30.2 (5.2) [152]	30.6 (5.6) [154]	.538
Mean days after vaccination of second postvaccination immunogenicity visit (SD)	94.0 (39.8) [293]	92.7 (42.9) [150]	95.5 (36.3) [143]	.551
Mean days after vaccination of third postvaccination immunogenicity visit (SD)	249.9 (37.8) [297]	248.2 (36.8) [149]	251.6 (38.9) [148]	.443
Mean days between first and second postvaccination immunogenicity visits (SD)^a^	71.4 (35.4) [241]	71.3 (39.5) [120]	71.4 (31.1) [121]	.983
Mean days between second and third postvaccination immunogenicity visits (SD)^a^	159.5 (20.7) [269]	157.6 (24.0) [138]	161.6 (16.3) [131]	.117
Delivery <37 wk gestational age, No. (12)	23 (7.9) [291]	13 (8.8) [147]	10 (6.9) [144]	.548
Median birth weight, kg, (range)	3.1 (1.5–4.8) [291]	3.1 (2.0–4.1) [147]	3.2 (1.5–4.8) [144]	.177

Numbers in brackets represent the number of participants with available information.

Abbreviations: IIV, trivalent inactivated influenza vaccine; SD, standard deviation.

^a^Only participants who had their scheduled visits at least 21 days apart.

At enrollment, HIV-infected women in the IIV group were younger than in the placebo group (mean age, 27.1 years vs 29.2 years; *P* = .009). Other demographic characteristics were similar between the 2 groups, including mean body mass index (28.7 kg/m^2^), mean gestational age at enrollment (27.2 weeks), percentage who had been pregnant before (73.6%), median CD4^+^ T-lymphocyte count (410 cells/µL), and percentage with undetectable HIV-1 RNA (24.2%) (Table 2). The follow-up of the HIV-infected women and availability of samples for HAI serology testing is illustrated in Supplementary Figure 1*B*.

**Table 2.** Baseline Demographic Characteristics for the Human Immunodeficiency Virus–Infected Cohort Participants and Scheduled Visits Time-points

Characteristic	Overall	IIV	Placebo	*P* Value
Mean age, y (SD)	28.1 (5.1) [166]	27.1 (4.9) [88]	29.2 (5.2) [78]	.009
Mean body mass index, kg/m^2^ (SD)	28.7 (5.2) [132]	29.0 (4.9) [71]	28.2 (5.5) [61]	.352
Mean gestational age, wk (SD)	27.2 (3.8) [166]	27.6 (.9) [88]	26.8 (3.7) [78]	.160
Nulliparous, No. (12)	34 (20.6) [165]	17 (19.5) [87]	17 (21.8) [78]	.721
Primigravida, No. (12)	27 (16.4) [165]	15 (17.2) [87]	12 (15.4) [78]	.748
Mean days after vaccination of first postvaccination immunogenicity visit (SD)	32.2 (7.9) [158]	32.2 (6.9) [83]	32.2 (9.0) [75]	.995
Mean days after vaccination of second postvaccination immunogenicity visit (SD)	93.0 (33.0) [149]	92.4 (37.2) [79]	93.6 (27.8) [70]	.823
Mean days after vaccination of third postvaccination immunogenicity visit (SD)	250.6 (38.0) [157]	249.5 (41.4) [84]	251.9 (33.8) [73]	.685
Mean days between first and second postvaccination immunogenicity visits (SD)^a^	64.7 (26.9) [126]	64.7 (28.5) [63]	64.7 (25.5) [63]	.984
Mean days between second and third postvaccination immunogenicity visits (SD)^a^	163.1 (26.6) [140]	162.5 (34.3) [75]	163.8 (13.3) [65]	.777
Median CD4^+^ count, cells/µL (IQR)	410 (287–565) [163]	410 (284–581) [87]	428 (307–561) [76]	.475
Women with CD4^+^ count <250 cells/µL, No. (12)	29 (17.8) [163]	15 (17.2) [87]	14 (18.4) [76]	
Women with CD4^+^ count 250–500 cells/μL, No. (12)	82 (50.3) [163]	46 (52.9) [87]	36 (47.4) [76]	.773
Women with CD4^+^ count >500 cells/μL, No. (12)	52 (31.9) [163]	26 (29.9) [87]	26 (34.2) [76]	
Women with HIV-1 RNA <40 copies/mL, No. (12)	39 (24.2) [161]	16 (18.6) [86]	23 (30.7) [75]	.075
Women on antiretroviral therapy, No. (12)	132 (79.5) [166]	70 (79.6) [88]	62 (79.5) [78]	.993
Delivery <37 wk gestational age, No. (12)	19 (12.4) [153]	10 (12.2) [82]	9 (12.7) [71]	.928
Median birth weight, kg (range)	3.0 (2.0–4.3) [153]	3.0 (2.1–4.3) [82]	2.9 (2.0–4.1) [71]	.341

Numbers in brackets represent the number of participants with available information.

Abbreviations: HIV-1, human immunodeficiency virus; IIV, trivalent inactivated influenza vaccine; IQR, interquartile range; SD, standard deviation.

^a^Only participants who had their scheduled visits at least 21 days apart.

### Vaccine Efficacy Against PCR-CI and/or SDI Among HIV-Uninfected Women

In HIV-uninfected placebo recipients, the incidence of PCR-CI (5.6%; 95% CI, 2.6%–10.4%) among women enrolled into the nested immunogenicity study was not significantly different from those not included (3.3%; 95% CI, 2.3%–4.8%; *P* = .161). The VE point-estimate for PCR-CI among HIV-uninfected women in the immunogenicity subset (66.9%; 95% CI, –20.1% to 90.9%) was similar to that in the full cohort (50.4%; 95% CI, 14.5%–71.2%; *P* = .07) [2].

Overall, 41.2% (7/24) of the SDI among IIV recipients and 52.6% (30/57) among placebo recipients occurred between 1 month postvaccination and within 7 days of delivery, whereas the remaining cases occurred thereafter up until 24 weeks postpartum (Supplementary Table 1). There was poor κ correlation for SDI compared with PCR-CI (occurring after the second immunogenicity visit) for A/H1N1pdm09 (κ = 0.10), A/H3N2 (κ = 0.03), B/Victoria (κ = 0.09), and overall influenza strains (κ = 0.04) among placebo recipients and among IIV recipients (κ = 0.07 for A/H3N2). Among placebo recipients, the sensitivity of serology for identifying PCR-CI against A/H1N1pdm09, A/H3N2, and B/Victoria was 100% of 1, 50% of 2, and 50% of 2, respectively, with the corresponding HAI titers prior to PCR-CI being 1:10, <1:10, and 1:20 in the women in whom serologic conversion was observed. Among the 2 IIV recipients who had PCR-CI for A/H3N2, only 1 had serologic response, with an HAI titer prior to PCR-CI of <1:10. Among the 3 participants with PCR-CI in whom no serologic conversion was observed, the time between the illness visit and earliest subsequent time-point for which convalescent plasma samples were available for serology testing was 49 and 181 days for A/H3N2 and 152 days for B/Victoria. The HAI titers in these women prior to PCR-CI were <1:10 and 1:10 in the placebo recipients and 1:10 in the IIV recipient.

Excluding the B/Yamagata cases (which was mismatched to the B/Victoria strain included in the seasonal IIV), the incidence among placebo recipients of at least 1 episode of SDI (35.0%) was 7.0-fold (95% CI, 3.4- to 14.2-fold) greater than PCR-CI (5.0%); and similarly so among IIV recipients (14.3% vs 1.9%; risk ratio [RR], 7.7; 95% CI, 2.3–25.0). Among placebo recipients, SDI was evident in 10.6% for A/H1N1pdm09, 23.1% for A/H3N2, and 10.6% for B/Victoria, compared to 3.1%, 12.4%, and 0.6%, respectively, among IIV recipients (Table 3). The overall VE estimate for SDI was 59.2% (95% CI, 37.0%–73.5%; *P* < .001), including being significant for the individual vaccine strains of A/H1N1pdm2009 (70.8%; *P* = .008), A/H3N2 (46.3%; *P* = .012), and B/Victoria (94.2%; *P* < .001) (Table 3). The NNT to prevent PCR-CI was 5.4-fold greater (27; 95% CI, 12–∞) than for prevention of SDI (NNT, 5; 95% CI, 3–9) or the composite of either (NNT, 5; 95% CI, 3–8).

**Table 3.** Efficacy of Trivalent Inactivated Influenza Vaccination of Human Immunodeficiency Virus–Uninfected Women up to 24 Weeks Postpartum

Maternal Efficacy Endpoint Outcome	2011 Cohort				2012 Cohort				Overall Cohort			
	IIV (n = 77)	Placebo (n = 82)	VE (95% CI)	*P* Value	IIV (n = 84)	Placebo (n = 78)	VE (95% CI)	*P* Value	IIV (n = 161)	Placebo (n = 160)	VE (95% CI)	*P* Value
PCR-CI including B/Yamagata	1 (1.3)	3 (3.7)	64.5 (–234.0 to 96.2)	.621	2 (2.4)	6 (7.7)	69.0 (–48.8 to 93.6)	.156	3 (1.9)	9 (5.6)	66.9 (–20.1 to 90.9)	.086
PCR-CI excluding B/Yamagata	1 (1.3)	2 (2.4)	46.8 (–475.4 to 95.1)	.999	2 (2.4)	6 (7.7)	69.0 (–48.8 to 93.6)	.156	3 (1.9)	8 (5.0)	62.7 (–37.9 to 89.9)	.138
Serologically diagnosed A/H1N1pdm09	1 (1.3)	13 (15.9)	91.8 (38.9–98.6)	.001	4 (4.8)	4 (5.1)	71.4 (–258.6 to 76.0)	.999	5 (3.1)	17 (10.6)	70.8 (22.7–89.0)	.008
Serologically diagnosed A/H3N2	5 (6.5)	17 (20.7)	68.7 (19.2–87.9)	.011	15 (17.9)	20 (25.6)	30.4 (–26.2 to 61.6)	.229	20 (12.4)	37 (23.1)	46.3 (11.6–67.4)	.012
Serologically diagnosed B/Victoria	1 (1.3)	14 (17.1)	92.4 (43.5–99.0)	.001	0	3 (3.8)	1	.109	1 (0.6)	17 (10.6)	94.2 (56.6–99.2)	<.001
Serologically diagnosed infection for at least 1 strain	7 (9.1)	32 (39.0)	76.7 (50.4–89.1)	<.001	16 (19.0)	24 (30.8)	38.1 (–75.6 to 64.4)	.084	23 (14.3)	56 (35.0)	59.2 (37.0–73.5)	<.001
Composite SDI or PCR-CI A/H1N1pdm09	1 (1.3)	13 (15.9)	91.8 (38.9–98.6)	.001	4 (4.8)	4 (5.1)	71.4 (–258.6 to 76.0)	.999	5 (3.1)	17 (10.6)	70.8 (22.7–89.0)	.008
Composite SDI or PCR-CI A/H3N2	6 (7.8)	17 (20.7)	62.4 (9.6–84.4)	.020	16 (19.0)	22 (28.2)	32.5 (–188.7 to 61.6)	.169	22 (13.7)	39 (24.5)	43.9 (9.9–65.1)	.015
Composite SDI or PCR-CI B/Victoria	1 (1.3)	14 (17.1)	92.4 (43.5–99.0)	.001	0	4 (5.1)	1	.052	1 (6.2)	18 (11.3)	94.5 (59.1–99.3)	<.001
Overall composite SDI or PCR-CI (including B/Yamagata)	8 (10.4)	32 (39.0)	73.4 (45.9–87.9)	<.001	17 (20.2)	27 (34.6)	41.5 (1.4–65.3)	.040	25 (15.5)	59 (36.9)	57.9 (36.3–72.1)	<.001
Overall composite SDI or PCR-CI (excluding B/Yamagata)	8 (10.4)	32 (39.0)	73.4 (45.9–87.9)	<.001	17 (20.2)	27 (34.6)	41.5 (1.4–65.3)	.040	25 (15.5)	59 (36.9)	57.9 (36.3–72.1)	<.001

Abbreviations: CI, confidence interval; IIV, trivalent inactivated influenza vaccine; PCR-CI, polymerase chain reaction–confirmed influenza illness; SD, standard deviation; SDI, serologically diagnosed influenza infection; VE, vaccine efficacy.

### Vaccine Efficacy Against PCR-CI and/or SDI Among HIV-Infected Women

Among HIV-infected women, overall, 75.0% (12/16) and 65.7% (23/35) of the SDI among IIV and placebo recipients, respectively, occurred between 1 month postvaccination and within 7 days of delivery, whereas the remaining cases occurred thereafter up until 24 weeks postpartum (Supplementary Table 2). There was poor κ correlation for SDI compared with PCR-CI (occurring at least 21 days postvaccination), overall (κ = 0.04), as well as specifically for A/H1N1pdm09 (κ = 0.20), A/H3N2 (κ = 0.08), and B/Victoria (κ = 0.18) among placebo recipients and among IIV recipients (κ = 0.04). Seventeen of the 20 PCR-CI cases (excluding non-vaccine-matched B/Yamagata cases) in HIV-infected women occurred after the second immunogenicity visit, among whom serologic conversion was observed in 61.5% (8/13) of placebo recipients and 25% (1/4) of IIV recipients (*P* = .29). The sensitivity of serology for identifying PCR-CI against A/H1N1pdm09, A/H3N2, and B/Victoria was 75% of 8, 25% of 4, and 100% of 1, respectively among placebo recipients, and 33.3% of 3 for A/H1N1pdm09 among IIV recipients. Among those with PCR-CI in whom no serologic conversion was observed, the mean time between the PCR-CI visit and earliest subsequent time-point for which convalescent plasma samples were available for serologic testing was 87.5 days (range, 7–153 days). The median HAI titers prior to PCR-CI in the 9 women who had a serologic response was 10 (interquartile range [IQR], 10–20) compared with 30 (IQR, 15–120) (*P* = .083) in the 8 women in whom no serologic conversion was observed.

The incidence of PCR-CI among the HIV-infected women included in this analysis was 20.5% in placebo recipients and 8.0% in IIV recipients, for a VE of 61.2% (95% CI, 10.7%–83.2%). Among placebo recipients, SDI by at least 1 of the 3 vaccine strains was evident in 43.6%, including 34.6% for A/H1N1pdm09, 14.1% for A/H3N2, and 11.5% for B/Victoria (Table 4). Serologic evidence of infection was lower among the IIV recipients overall (17.0%; VE, 60.9%; *P* < .001), as well as specifically for A/H1N1pdm09 (13.6%; VE, 60.6%; *P* = .002), A/H3N2 (3.4%; VE, 75.8%; *P* = .022), and B/Victoria (6.8%; VE, 40.9%; *P* = .29) (Table 4). The incidence of at least 1 episode of SDI (43.6%) was 2.6-fold (95% CI, 1.5- to 4.6-fold) greater than PCR-CI (excluding B/Yamagata; 16.7%) among HIV-infected placebo recipients, and similarly so among the IIV recipients (17.0% vs 8.0%, respectively; RR, 2.1; 95% CI, .9–5.0) (Table 4).

**Table 4.** Efficacy of Trivalent Inactivated Influenza Vaccination of Human Immunodeficiency Virus–Infected Women up to 24 Weeks Postpartum, 2011

Maternal Efficacy Endpoint Outcome	IIV (n = 88)	Placebo (n = 78)	VE (95% CI)	*P* Value
PCR-CI including B/Yamagata	7 (8.0)	16 (20.5)	61.2 (10.7–83.2)	.019
PCR-CI excluding B/Yamagata	7 (8.0)	13 (16.7)	52.3 (−135.6 to 79.9)	.085
Serologically diagnosed A/H1N1pdm09	12 (13.6)	27 (34.6)	60.6 (27.7–78.5)	.002
Serologically diagnosed A/H3N2	3 (3.4)	11 (14.1)	75.8 (16.5–93.0)	.022
Serologically diagnosed B/Victoria	6 (6.8)	9 (11.5)	40.9 (−58.6 to 78.0)	.290
Serologically diagnosed infection for at least 1 strain	15 (17.0)	34 (43.6)	60.9 (33.9–76.9)	<.001
Composite SDI or PCR-CI A/H1N1pdm09	17 (19.3)	31 (39.7)	51.4 (19.3–70.7)	.004
Composite SDI or PCR-CI A/H3N2	4 (4.5)	13 (16.7)	72.7 (19.8–90.7)	.019
Composite SDI or PCR-CI B/Victoria	6 (6.8)	9 (11.5)	40.9 (−58.6 to 78.0)	.290
Overall composite SDI or PCR-CI (including B/Yamagata)	20 (22.7)	42 (53.8)	57.8 (34.7–72.7)	<.001
Overall composite SDI or PCR-CI (excluding B/Yamagata)	20 (22.7)	40 (51.3)	55.7 (31.1–71.5)	<.001

Abbreviations: CI, confidence interval; IIV, trivalent inactivated influenza vaccine; PCR-CI, polymerase chain reaction–confirmed influenza illness; SDI, serologically diagnosed influenza infection; VE, vaccine efficacy.

The VE estimates for the composite endpoint of either PCR-CI or SDI (57.8%; 95% CI, 34.7%–72.7%), was similar to that for PCR-CI alone (61.2%) or only SDI (60.9%) (Table 4). The NNT to prevent 1 episode of SDI (4; 95% CI, 3–8) was 2-fold lower than PCR-CI (8; 95% CI, 4–52), albeit with overlap of the 95% CI.

In HIV-infected women, being on antiretroviral therapy at enrollment and CD4^+^ T-lymphocyte count were not associated with rate of either PCR-CI or SDI.

## DISCUSSION

In contrast to previous reports that serologic endpoints might overestimate seasonal IIV VE [7–9], the results from our randomized placebo-controlled trial in pregnant women yielded similar point-estimates of VE using a serologic endpoint of HAI serologic conversion compared to PCR-CI (59.2% vs 62.7%, respectively) in HIV-uninfected and HIV-infected women (60.9% vs 52.3%, respectively) for vaccine-matched strains. Also, expanding the VE endpoint to include SDI resulted in significant difference in the NNT to prevent a single case of influenza virus infection among the women enrolled. Among HIV-uninfected women, this ratio changed 5.4-fold from 27:1 for PCR-CI to 5:1 for the composite (or SDI alone), whereas among HIV-infected women there was a 2-fold difference (changing from 8:1 to 4:1). Notably, the difference in NNT to prevent 1 case of influenza virus infection in HIV-uninfected compared with HIV-infected women was less marked when using SDI as an endpoint (5 vs 4) than for PCR-CI (27 vs 8). Also, the incidence of at least 1 episode of SDI was similar in 2011 between HIV-uninfected (39.0%) and HIV-infected (43.6%) placebo recipients, despite a 7-fold higher incidence of non-B/Yamagata PCR-CI in HIV-infected (16.7%) compared with HIV-uninfected women (2.4%; *P* = .002). This indicates greater susceptibility to developing a clinically important illness from influenza virus that involved a medical visit in HIV-infected compared with HIV-uninfected women, which was likely independent of community exposure to the virus.

The use of serology in our study provided a more sensitive measure of seasonal influenza exposure compared to that identified by PCR-CI, including a 5-fold greater risk of SDI in HIV-uninfected women and a 2-fold greater risk among HIV-infected women. Although many of the SDI cases were not identified as PCR-CI, despite our weekly active surveillance, it is conceivable that such subclinical or asymptomatic influenza virus infection could be of clinical relevance. Included in this is the possibility of subclinical influenza virus infection eliciting an occult immune response, which could affect the well-being of the fetus and increase susceptibility to adverse birth outcomes such as prematurity and stillbirth, as has been reported by others in large observational studies [16–20], or result in increased transmission of influenza within a community with high rates of comorbid conditions. Nonetheless, we did not observe any difference in fetal outcomes of premature birth or stillbirths in our randomized controlled trial [2], and others have called into question whether any association would be of sufficient magnitude to be detectable through most cohort study designs [21].

The limited sample size of our study for PCR-CI, for which we did not observe any difference in serologic conversion rates between IIV and placebo recipients, limits our ability to corroborate whether serologic responses to influenza virus infection are attenuated among IIV recipients, as has been previously suggested [7]. Although not significant, in HIV-infected women, there was a trend for women with higher HAI titers prior to PCR-CI not achieving serologic conversion. Overall seroconversion rates for PCR-CI cases were 57.1% among HIV-uninfected and 52.9% in HIV-infected women, suggesting that not all cases of influenza virus infection were identified by serology in our study and that serology itself could underestimate seasonal influenza exposure. This could be due to an attenuated antibody response to natural infection, or that the timing of availability of plasma postinfection in our study was inadvertently far removed from when the infection occurred, as was evident in some cases, and that there had been natural waning of antibody over time [22].

Limitations of this study include that we did not have samples for serologic testing among the full cohort of HIV-uninfected women and we were only able to analyze the comparative VE against PCR-CI and SDI among a nested subset of participants. Although this reduced the number of PCR-CI included in the comparative analysis, the VE point estimate for PCR-CI was similar between those included in the immunogenicity subset and the overall study population. Also, while the incidence of SDI was higher than for PCR-CI, it is possible that HAI serology is not specific for true influenza virus infection [23]. Such false-positive results could have overestimated the incidence of SDI in our study, albeit unlikely to the magnitude of difference observed for incidence of PCR-CI compared with SDI among HIV-uninfected placebo recipients. Furthermore, among HIV-uninfected women, as we only had breakthrough cases of A/H3N2 among IIV recipients, we were unable to analyze for differences in serologic conversion rates between IIV and placebo recipients for infection by A/H1N1pdm09 or B/Victoria.

In conclusion, although the use of serologic assays might have limitations in the clinical evaluation of IIV efficacy, including measuring a high burden of infections that might not be clinically evident, it nevertheless provides a more detailed evaluation of exposure to seasonal influenza virus and efficacy of IIV in preventing both PCR-CI and, possibly, subclinical influenza virus infection.

## Supplementary Data

Supplementary materials are available at *Clinical Infectious Diseases* online. Consisting of data provided by the authors to benefit the reader, the posted materials are not copyedited and are the sole responsibility of the authors, so questions or comments should be addressed to the corresponding author.

## Supplementary Material

Suppl_Figure_17Feb2017

SupplementaryTables_cid

## Notes

***Acknowledgments.*** The authors thank Niteen Wairagkar, program officer acting on behalf of the Bill & Melinda Gates Foundation; all of the study participants, the staff of the Departments of Obstetrics, Neonatology, and Paediatrics at Chris Hani Baragwanath Academic Hospital, Soweto, South Africa, for their dedication to their patients, including our trial participants; and the study midwives, nurses, laboratory staff, counsellors, and data capturers.

***Financial support.*** This study was supported by the Bill & Melinda Gates Foundation (grant number OPP1002747); the South African Research Chairs Initiative of the Department of Science and Technology and National Research Foundation in Vaccine Preventable Diseases; and the Medical Research Council, Respiratory and Meningeal Pathogens Research Unit.

***Potential conflicts of interest.*** J. R. O. is an employee of the World Health Organization. All other authors report no potential conflicts. All authors have submitted the ICMJE Form for Disclosure of Potential Conflicts of Interest. Conflicts that the editors consider relevant to the content of the manuscript have been disclosed.
