# Supplementary material for: Contribution of Serologic Assays in the Evaluation of Influenza Virus Infection Rates and Vaccine Efficacy in Pregnant Women: Report From Randomized Controlled Trials
Source: Clin Infect Dis. 2017 Mar 21;64(12):1773–9. doi: 10.1093/cid/cix241 (PMC5447878; doi:10.1093/cid/cix241)
Supplement: Suppl_Figure_17Feb2017 [file cix241_suppl_Suppl_Figure_17Feb2017.docx]

Figure 1a. HIV-uninfected women enrolled into the study and availability of samples for serology testing

**Inactivated influenza vaccine Placebo**

Allocated to and received placebo,

**n=1054**

Allocated to and received IIV3,

**n=1062**

Co-enrolled in immunogenicity subset,

**n=188**

Co-enrolled in immunogenicity subset,

**n=188**

Women with serology results post-vaccination,

**n=176**

Women with serology results post-vaccination,

**n=177**

Missed visits, n=2

Withdrawals, n=8

Lost-to follow-up, n=2

Missed visits, n=6

Withdrawals, n=5

Women with at least two immunogenicity visits post-vaccination, and included in the analysis

**n=161**

Women with at least two immunogenicity visits post-vaccination, and included in the analysis

**n=160**

Missed visits, n=7

Withdrawals, n=3

Lost-to follow-up, n=5

Missed visits, n=10

Withdrawals, n=1

Lost-to follow-up, n=6

Figure 1b. HIV-infected women enrolled into the study and availability of samples for serology testing

**Inactivated influenza vaccine Placebo**

Allocated to and received IIV3,

**n=100**

Allocated to and received placebo,

**n=94**

Women with serology results post-vaccination,

**n=96**

Women with serology results post-vaccination,

**n=177**

Missed visits, n=2

Withdrawals, n=1

Lost-to follow-up, n=1

Missed visits, n=5

Withdrawals, n=3

Lost-to follow-up, n=4

Women with at least two immunogenicity visits post-vaccination, and included in the analysis

**n=88**

Women with at least two immunogenicity visits post-vaccination, and included in the analysis

**n=78**

Withdrawals, n=5

Lost-to follow-up, n=3

Missed visits, n=1

Withdrawals, n=3

Lost-to follow-up, n=2
