# Supplementary material for: Contribution of Serologic Assays in the Evaluation of Influenza Virus Infection Rates and Vaccine Efficacy in Pregnant Women: Report From Randomized Controlled Trials
Source: Clin Infect Dis. 2017 Mar 21;64(12):1773–9. doi: 10.1093/cid/cix241 (PMC5447878; doi:10.1093/cid/cix241)
Supplement: SupplementaryTables_cid [file cix241_suppl_SupplementaryTables_cid.docx]

**Supplementary Table 1.** Serologically diagnosed influenza infection in HIV-uninfected women during the two study periods where serological assessment was performed

| Strain specific serological diagnosed influenza infection | Between post-vaccination visit and delivery visit | | | Between delivery visit and last visit | | |
| --- | --- | --- | --- | --- | --- | --- |
|  | IIV3 | Placebo | Overall | IIV3 | Placebo | Overall |
| Serologically-diagnosed infection for A/(H1N1)pdm09 | 1 | 13 | 14 | 4 | 3^1^ | 7 |
| Serologically-diagnosed infection for A/H3N2 | 5 | 15 | 20 | 15 | 19^2^ | 34 |
| Serologically-diagnosed infection for B/Victoria | 1 | 14 | 15 | 0 | 2^3^ | 2 |
| Serologically-diagnosed infection for at least one strain | 7 | 30 | 37 | 17^5^ | 27^4^ | 44 |

^1^One participant missed delivery visit but had a 4-fold rise for A/H1N1pdm09 between post-vaccination visit and last visit

^2^Three participants missed delivery visit but had a 4-fold rise for A/H3N2 between post-vaccination visit and last visit

^3^One participant missed delivery visit but had a 4-fold rise for A/H1N1pdm09 between post-vaccination visit and last visit

^4^Four participants missed delivery visit but had a 4-fold rise for at least one of the strains between post-vaccination visit and last visit. One participant had a 4-fold rise for at least one of the strains between both time points

^5^One participant had a 4-fold rise for at least one of the strains between both time points

**Supplementary Table 2.** Serologically diagnosed influenza infection in HIV-infected women during the two study periods where serological assessment was performed

| Strain specific serological diagnosed influenza infection | Between post-vaccination visit and delivery visit | | | Between delivery visit and last visit | | |
| --- | --- | --- | --- | --- | --- | --- |
|  | IIV3 | Placebo | Overall | IIV3 | Placebo | Overall |
| Serologically-diagnosed infection for A/(H1N1)pdm09 | 9 | 21 | 30 | 3 | 6^1^ | 9 |
| Serologically-diagnosed infection for A/H3N2 | 2 | 3 | 5 | 1 | 8^2^ | 9 |
| Serologically-diagnosed infection for B/Victoria | 6 | 6 | 12 | 0 | 3 | 3 |
| Serologically-diagnosed infection for at least one strain | 12 | 23 | 35 | 4^3^ | 12^4^ | 16 |

^1^Two participants missed delivery visit but had a 4-fold rise for A/H1N1pdm09 between post-vaccination visit and last visit

^2^Two participants missed delivery visit but had a 4-fold rise for A/H3N2 between post-vaccination visit and last visit

^3^One participant had a 4-fold rise for at least one of the strains between both time points

^4^Two participants missed delivery visit but had a 4-fold rise for at least one of the strains between post-vaccination visit and last visit. One participant had a 4-fold rise for at least one of the strains between both time points
